# Miscellany

**Published:** 1905-12

**Authors:** 


### MISCELLANY.

---

### Epilepsy Prize.

At the Fifth Annual Meeting of TheNational Association for the Study of Epilepsy, held in the Academy of Medicine, New York City on November 29, 1905, Dr. W. P. Spratling, president, announced that the Association offered a prize of $300 for the best essay on the etiology of epilepsy.

Physicians in any country may compete for this prirze. The award will be made in November, 1906, but all essays submitted must be sent in by September 1st of that year.

Details as to conditions governing the award may be obtained from Dr. Spratling, Superintendent of the Craig Colony for Epileptics, Sonyea, Livingston County, N. Y.

---

The State Civil Service Commission announces examinations to be held on January 13, 1906, for the following positions in the state and county service: abstract clerk, Onondaga County Clerk's office; assistant in microscopy, Cancer Laboratory, Buffalo, $720; carpenter, State Industrial School, Rochester, $50 a month; steam engineer and assistant in State hospitals, departments and institutions in the county service of Albany, Erie, Monroe, Onondaga and Westchester counties; foreman of fish hatchery, $1,080; inspector of records and accounts, State Board of Charities, $1,200 to $1,400; matron, Craig Colony, $720 to $900; milk expert, Department of Agriculture, $800 to $1,000; male officer, State institutions, $540; woman industrial teacher, State Custodial Asylum, Newark, $360 and maintenance; physical instructor, State institutions, $540 to $1200; sanitary agent, Department of Agriculture, $5 a day. The last day for filing applications is January 8th. Application forms and detailed information may be obtained by addressing the Chief Examiner of the Commission at Albany.

